# PRC2-independent actions of H3.3K27M in embryonic stem cell differentiation

**DOI:** 10.1093/nar/gkac800

**Published:** 2022-09-26

**Authors:** Lea R Z Cohen, Binyamin Kaffe, Eden Deri, Chen Leibson, Malka Nissim-Rafinia, Moria Maman, Nofar Harpaz, Guy Ron, Efrat Shema, Eran Meshorer

**Affiliations:** Department of Genetics, The Alexander Silberman Institute of Life Sciences, The Hebrew University of Jerusalem, Jerusalem 9190401, Israel; The Edmond and Lily Safra Center for Brain Sciences (ELSC), The Hebrew University of Jerusalem, Jerusalem 9190401, Israel; Department of Genetics, The Alexander Silberman Institute of Life Sciences, The Hebrew University of Jerusalem, Jerusalem 9190401, Israel; Department of Genetics, The Alexander Silberman Institute of Life Sciences, The Hebrew University of Jerusalem, Jerusalem 9190401, Israel; The Edmond and Lily Safra Center for Brain Sciences (ELSC), The Hebrew University of Jerusalem, Jerusalem 9190401, Israel; Department of Genetics, The Alexander Silberman Institute of Life Sciences, The Hebrew University of Jerusalem, Jerusalem 9190401, Israel; Department of Genetics, The Alexander Silberman Institute of Life Sciences, The Hebrew University of Jerusalem, Jerusalem 9190401, Israel; Department of Genetics, The Alexander Silberman Institute of Life Sciences, The Hebrew University of Jerusalem, Jerusalem 9190401, Israel; Department of Immunology and Regenerative Biology, Weizmann Institute of Science, Rehovot 76100, Israel; The Racah Institute of Physics, The Center for Nanoscience and Nanotechnology, The Hebrew University, Jerusalem 9190401, Israel; Department of Immunology and Regenerative Biology, Weizmann Institute of Science, Rehovot 76100, Israel; Department of Genetics, The Alexander Silberman Institute of Life Sciences, The Hebrew University of Jerusalem, Jerusalem 9190401, Israel; The Edmond and Lily Safra Center for Brain Sciences (ELSC), The Hebrew University of Jerusalem, Jerusalem 9190401, Israel

## Abstract

The histone H3 variant, H3.3, is localized at specific regions in the genome, especially promoters and active enhancers, and has been shown to play important roles in development. A lysine to methionine substitution in position 27 (H3.3K27M) is a main cause of Diffuse Intrinsic Pontine Glioma (specifically Diffuse Midline Glioma, K27M-mutant), a lethal type of pediatric cancer. H3.3K27M has a dominant-negative effect by inhibiting the Polycomb Repressor Complex 2 (PRC2) activity. Here, we studied the immediate, genome-wide, consequences of the H3.3K27M mutation independent of PRC2 activity. We developed Doxycycline (Dox)-inducible mouse embryonic stem cells (ESCs) carrying a single extra copy of WT-H3.3, H3.3K27M and H3.3K27L, all fused to HA. We performed RNA-Seq and ChIP-Seq at different times following Dox induction in undifferentiated and differentiated ESCs. We find increased binding of H3.3 around transcription start sites in cells expressing both H3.3K27M and H3.3K27L compared with WT, but not in cells treated with PRC2 inhibitors. Differentiated cells carrying either H3.3K27M or H3.3K27L retain expression of ESC-active genes, in expense of expression of genes related to neuronal differentiation. Taken together, our data suggest that a modifiable H3.3K27 is required for proper histone incorporation and cellular maturation, independent of PRC2 activity.

## INTRODUCTION

The epigenetic code has been extensively studied in recent years utilizing genome-wide approaches, resulting in comprehensive mapping of histone modifications in multiple cell types across the genome (1,2). Apart from being post-translationally modified, the four replication-dependent core histones, which comprise the nucleosome (H2A, H2B, H3 and H4), can all be replaced by a number of replication-independent variants of varying functions, binding preferences, and interactions (3–6). Among these is the histone variant H3.3, which plays important roles in differentiation and development (7–9). Despite its almost complete identity to the canonical H3.1, differing by only 4 amino acids, its genome-wide pattern is distinct from that of H3.1. H3.3 is enriched in both telomeric heterochromatin and active euchromatin, where it is preferentially incorporated by the histone chaperone HirA in promoters and enhancers (10–12). H3.3 also shows differential turnover at different genomic sites, and higher H3.3 turnover was reported in active regions in a cell-type specific manner (13–15). In particular, H3.3 turnover plays important roles in neurons, where it mediates specific activity-dependent patterns of gene expression (16). In embryonic stem cells (ESC), a set of genes is marked by the active chromatin mark H3K4me3 as well as the suppressive H3K27me3 mark. This bivalency state which is found in locations with H3.3 deposition (11) is important for ESC differentiation and development (17,18). Disruption of H3K27 tri-methylation by a K27R mutation causes a severe defect in development (19).

H3.3 attracted much attention recently when it was discovered that a mutation in its K27 residue (H3.3K27M) is present in a striking percentage (∼80%, together with H3.1K27M) of pediatric Diffuse Intrinsic Pontine Gliomas (DIPG) (20–26). It is also considered a driver mutation in other types of cancers including Osteosarcoma, Acute myeloid leukemia, Posterior Fossa ependymoma and Pons/Brainstem gliomas (27–31). DIPG, found in young children, has an epigenetic profile which differs from that of adult glioblastomas (32). This, together with the fact that the expression profile of this deadly glioma shows patterns of early developmental stages (32), highlights the importance of the H3.3K27 residue during both development and cancer. Due to the unique properties and spread of the H3.3K27M (and H3.1K27M) mutations, it received a new classification group named Diffuse Midline Glioma (DMG), referring to diffused tumors with these mutations in midline brain areas (33).

The mutated H3.3 usually represents a relatively small fraction of all H3 in the cell, of <10% (34). H3.3K27M was found to have a dominant-negative effect by inhibiting the enzymatic activity of the Polycomb Repressor Complex 2 (PRC2), resulting in genome-wide depletion of H3K27me3 (35). Other mutations such as H3.3K27L do not inhibit the PRC2 complex and do not result in a genome wide reduction of H3K27me3 (35). At least in mice, the H3.3K27M mutation alone is sufficient for the induction of cancer (36), although in humans it is usually found together with mutations in other genes such as *Tp53*, *Atrx* and *Pdgfra* (37,38). Seeking downstream consequences, a recent study suggested that H3.3K27M have enriched interactions with histone chaperones (39).

In this work, we generated a Dox-inducible system that allowed us to study the immediate effects of H3.3 mutations. We analyzed the earliest consequences of ectopically expressing WT-H3.3, H3.3K27M or H3.3K27L on gene expression, H3.3 incorporation and histone modifications, in ESCs and during differentiation. We report that both H3.3K27M and H3.3K27L induce an H3.3-incorporation-related transcriptional signature of delayed differentiation, suggesting that the early epigenetic and transcriptional response to H3.3K27M is PRC2-independent.

## MATERIALS AND METHODS

### Cell line generation

Plasmids were constructed using Gibson. Mutations were inserted by site directed mutagenesis. KH2 mouse ESCs were transfected using Neon transfection system (Thermo-Fisher). Plasmids were transformed into mouse KH2 cells together with a Flipase vector. Colonies were selected by hygromycin selection.

### Cell culture

Cells were cultured on gelatin-coated tissue culture plates in ESC media (DMEM supplemented with 10% fetal calf serum, 100 U/ml penicillin, 100 mg/ml streptomycin, 2 mmol/l l-glutamine, 5 mg/ml MEM non-essential amino acids, 0.12 mmol/l β-mercaptoethanol and 1000 U/ml leukemia inhibitory factor) supplemented with 2i inhibitors cocktail (mitogen activated protein kinase (MAPK)/extracellular-signal-regulated kinase (ERK) kinase (MEK) inhibitor PD0325901 and the glycogen synthase kinase 3 (GSK3) inhibitor CHIR99021). For retinoic acid (RA) induced differentiation, cells were grown on gelatin-coated dishes for 4 days in ESC media without LIF/2i in the presence of 1 μM RA. All cells were cultured at 37°C in 5% CO_2_. For OPC differentiation, cells were differentiated according to (40), with a few adjustments. Briefly, ESC colonies were trypsinized (TrypLE) into single cells and passed through a 70 μm strainer, suspended in knockout serum replacement (KSR) medium, and transferred to 100mm petri dishes at a density of 50 000 cells/cm^2^ allowing to form embryoid bodies (EBs). From day 4 to day 7, RA (0.2 M) and purmorphamine (1 M) were added. At day 8, EBs were disaggregated and plated on 0.002% polyornithine-coated dishes in OPC medium for up to 30 days. The medium was changed every other day. The cells were trypsinized and replated when they become confluent (typically once).

### Immunofluorescence, cell extraction and Western blots

Cells were grown on cover-slips, fixed in 4% paraformaldehyde (15 min, room temp), washed (3× PBS, 5 min), permeabilized (0.5% Triton X-100, 5 min) and incubated with primary antibodies (1 hr, room temp or 4°C overnight) in 10% serum in PBS. Cells were washed (3× PBS, 5 min, room temp), incubated with secondary antibodies (Alexa488 / Alexa568, Molecular Probes, 1:1000, 1 h, room temp) in 10% serum in PBS, washed (3× PBS, 5 min, room temp), DAPI stained, washed (PBS) and mounted on microscope slides using anti-fade (Dako, Glostrup, Denmark). For whole cell extraction, cells were trypsinized, washed with cold PBS, centrifuged (500 *g*, 4°C, 5 min), resuspended in ice-cold hypotonic Lysis Buffer (20 mM Tris–Cl pH 7.5, 0.2 mM EDTA, 0.5 mM DTT, and 1:100 protease inhibitors cocktail [Sigma]), incubated on ice (10 min) with a similar volume of High Salt Buffer (20 mM Tris–Cl pH 7.5, 0.2 EDTA mM, 0.5 mM DTT, 1 M NaCl), centrifuged (20 000 *g*, 4°C, 30 min), supernatants collected, and protein concentration was determined using Bradford. Detection for WB was done using α-mouse, α-rabbit or α-goat antibodies conjugated to HRP (115-035-062, 111-035-144, 705-035-147 Jackson ImmunoResearch, respectively).

### Cell cycle analysis

Cells were trypsined to achieve single cells, and were incubated for 30 min in ESC media with Hoechst33342. Cells were then filtered and analyzed using CellStream Analyzer (Merkel) and FlowJo.

### Chromatin immunoprecipitation (ChIP)

Multiplexed Chromatin immunoprecipitation followed by next generation sequencing (MINT-ChIP-seq) was performed as previously described (41) using the following antibodies: α-HA (ab9110), α-H3 (Active-Motif), α-H3K27me3 (Millipore, 07-449) and αH3K27ac (a gift from Hitoshi Kimura, Osaka University). Sequencing was performed in-house using Illumina NextSeq500.

### RNA extraction, library preparation and sequencing

RNA was prepared using RNeasy (Qiagen) according to the manufacturer's instructions. Library preparation was done using QuantSeq 3′-mRNA-Seq Library Prep Kit (Lexogen) or KAPA mRNA-seq kit (Roche). Libraries were subjected to single-end sequencing using Illumina NextSeq500.

### ChIP-seq

FASTQ files were aligned using Bowtie2 (42) using default parameters. Resulting BAM files were sorted using SAMtools and duplicates were removed (43). Bedgragh files were made using BEDTools (44) and the reads normalized to RPM. For calculating the change in global H3K27Me3 and H3K27ac total read count between modification and H3.3 was calculated, and the change from early time points (4 and 8) to 72 h was calculated. Further analysis of our mESCs was done in MATLAB. In short, data from Bed files was loaded and (apart of chromosome 8) we renormalized the total number of reads to mapped reads. We analyzed the genome in resolution of 200 bp. Gene location data was taken from UCSC table browser and used for calculating Heatmaps, H3.3 profiles around TSS and genic distribution of H3.

Plots of H3.3 metadata from Human cell (45), were made using deepTools2 (46).

### RNA-seq analysis

FASTQ files were aligned to the mouse reference genome (mm9) using STAR (version 2.7.1) allowing up to 10 mismatches. The output was normalized to transcripts per million (TPM). Counts files were made. For genes with few variants, the expression level was accounted as the expression of the variant with the maximal expression. Although e-karyotyping did not detect any abnormalities, cytogenetic karyotyping of our cells revealed a potential duplication in chromosome 8 within all our clones. Therefore, to ensure duplication-based bias, chromosome 8 was excluded from all analyses.

### Differentially expressed genes

Differential expression analysis was performed on the raw expression counts using the R package DESeq2 (version 1.28.1) as well MATLAB. Experiments were repeated weeks apart for biological replicates. Genes with *P*-adjusted value <0.05 and fold-change <0.5 or >2 were marked as differentially expressed genes (DE genes). The DE genes were intersected with 1,304 genes known as related to glioblastoma downloaded from the curated CTD Gene-Disease Associations dataset (https://maayanlab.cloud/Harmonizome/gene_set/Glioblastoma/CTD+Gene-Disease+Associations) (47). Gene Ontology (GO) analysis was performed using Panther (48), and result were analyzed in MATLAB.

### CyTOF sample preparation

Cells were stained with antibodies targeting histone modifications and core histone proteins, run on CyTOF and analyzed as previously reported (49). In short, cells were harvested to a single cell suspension in PBS buffer. Cells’ live dead staining was performed by 1 min incubation with Cell-ID Cisplatin (Fluidigm) at a concentration of 1.25 μM, followed by rinses with DMEM + 10% FBS and MaxPar Cell Staining Buffer (Fluidigm). Cells were then fixed by 30 min incubation with nuclear antigen staining working solution (Maxpar Nuclear Antigen Staining Buffer Set, Fluidigm). Cells were rinsed with antigen permeabilization buffer (Maxpar Nuclear Antigen Staining Buffer Set, Fluidigm), and barcoded by 1 h incubation with Palladium-based barcodes (Cell-ID 20-Plex Pd Barcoding Kit, Fluidigm). Cells were rinsed twice with nuclear antigen permeabilization. Samples that run together were pooled together, where the same number of cells were taken from each sample. Cells were blocked for 10 min with NGS 10% (NGS, Cell Signaling Technology) and then incubated with antibodies pool for 30 min. Antibodies included were H3K27M antibody (ab240310, abcam) which was conjugated to 169Tm metal (201169A, Fluidigm), H3K27me3 antibody (61017, Active Motif) was conjugated to 168Er metal (201168B, Fluidigm), SOX2-150Nd antibody (3150019B, Fluidigm), H3-115ln antibody (711501, IonPath), H3.3 antibody (ab208690, abcam) was conjugated to 155Gd metal (201155A, Fluidigm), H4 antibody (ab238663, abcam) conjugated to 159Tb (201159A, Fluidigm). Cells were then rinsed twice with staining buffer, and fixed overnight in 10% formalin. The following day, DNA was labeled with Cell-ID Intercalator-Iridium (Fluidigm) which was added to the formalin solution to achieve a final concentration of 125 nM. After 30 min incubations, cells were rinsed twice with staining buffer and two more rinse Maxpar Cell Acquisition Solution (Fluidigm). Finally, cells were resuspended in cell acquisition solution containing 1:10 dilution of EQ Four Element Calibration Beads (Fluidigm), filtered through a 35 μm mesh cell strainer (Falcon) prior to acquisition on a Fluidigm Helios CyTOF system.

### CyTOF data processing analysis

CyTOF data was processed and analyzed as previously described (49). In short, data was normalized using the CyTOF software (Fluidigm), and then gated to live single cells using the Cytobank platform (Beckman Coulter) with the following: cisplatin 195Pt, iridium DNA label in 193Ir, event length, and the Gaussian parameters of width, center, offset and residual channels. Additionally, SOX2-150Nd was used to distinguish the mouse embryonic stem cells from the irradiated MEF cells they were grown with. Last, sample de-barcoding was performed using the CyTOF software. The processed data underwent arcsine transformation and standardization, and epigenetic markers were normalized to the core histone levels as was previously reported (49).

### Statistical analyses

Experiments, both RNA-seq and ChIP-seq, were performed in two biological replicates, weeks apart. In RA treated cells, we pooled the 24 and 72 h time-points, which were similar. For correlation analyses, we used Spearman correlation (of mean per gene of the area –1.2 kb to –200 b and 400 b to 1.2 kb from TSS). *P*-value was calculated using two-tailed Student's *t*-test, or *U*-test when comparing groups of different size, unless otherwise indicated.

### Databases used

https://genome.ucsc.edu/cgi-bin/hgTables

http://pantherdb.org/

https://maayanlab.cloud/Harmonizome/gene_set/Glioblastoma/CTD+Gene-Disease+Association

## RESULTS

### H3.3 mutations have little effect on H3.3 binding targets

To study the immediate consequences of H3.3K27M, we generated Doxycycline (Dox)-inducible KH2 FLIP-in mouse ESCs expressing a single extra copy of WT, K27M or K27L SNAP-HA-H3.3 (50). H3.3K27M was previously shown to have a dominant-negative effect by inhibiting the PRC2 complex, leading to an overall reduction in H3K27me3 (35). Therefore, to distinguish between the dominant-negative effects of H3.3K27M and effects in *cis* due to the missing lysine at position 27, we added the H3.3K27L mutant, which cannot be modified but has no global effect on PRC2 activity (35). The clones were sequenced, and Dox-induced expression was verified by qPCR, Western blot, and immunofluorescence (Supplementary Figure S1A–C). Dox induction of the mutations did not alter cell cycle kinetics in these cells (Supplementary Figure S1D).

**Figure 1.**
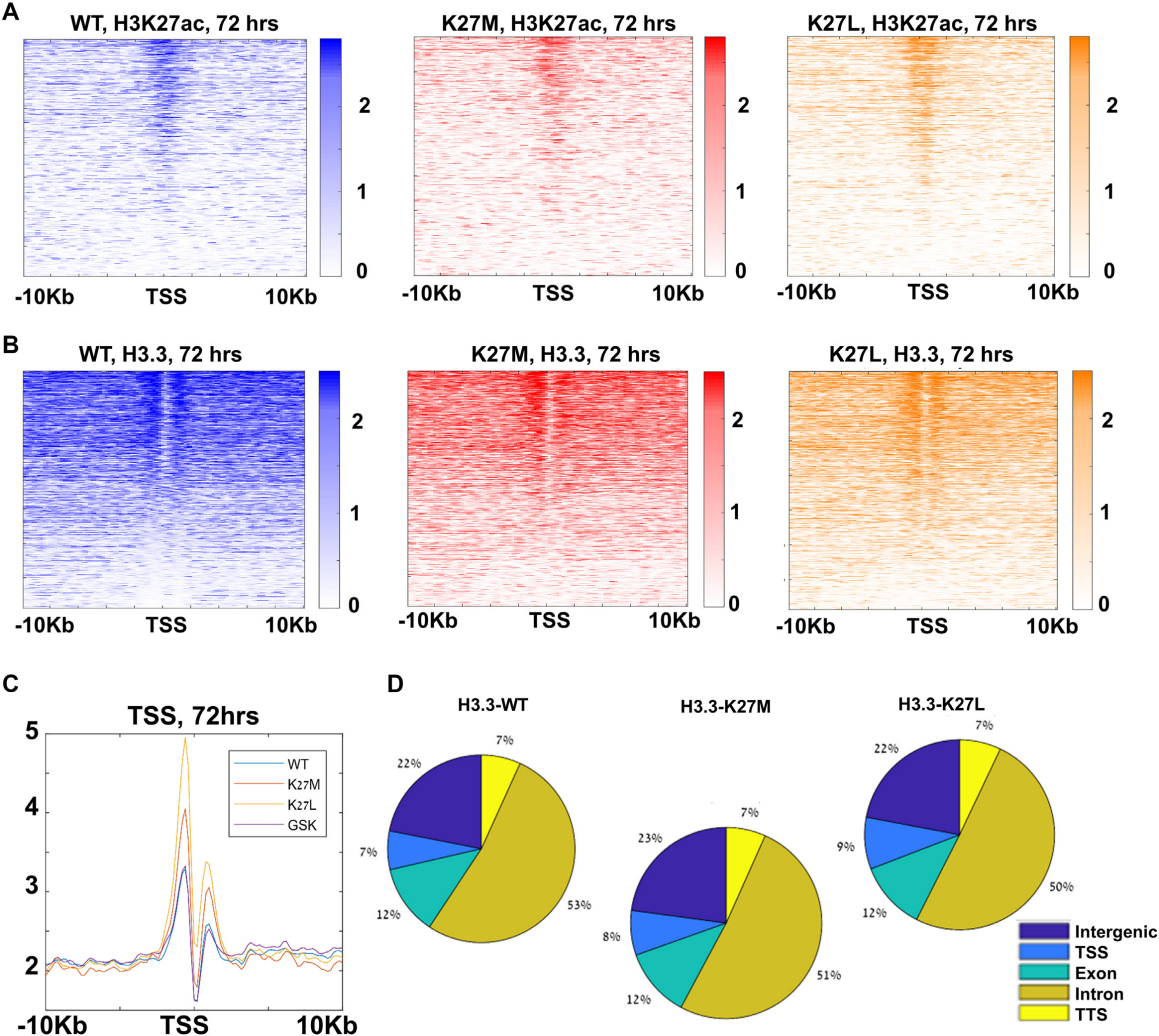
H3.3K27M/L mutations have little effect on H3.3 genome-wide distribution. (**A**) Heatmaps of H3K27ac around TSSs (±10 kb), at 72 h after Dox, in ESCs expressing WT H3.3 (blue), H3.3K27M (red) or H3.3K27L (orange). Genes are sorted by H3.3 binding in WT. (**B**) Heatmaps of H3.3-HA around TSSs (±10 kb), at 72 h after Dox, in ESCs expressing WT H3.3 (blue), H3.3K27M (red) or H3.3K27L (orange). Genes are sorted by H3.3 binding in WT. (**C**) H3.3 incorporation profile around TSSs 72 h after Dox. ESCs expressing H3.3K27M (red) or H3.3K27L (orange) show increased H3.3 binding in TSSs, not observed in ESCs treated with the PRC2 inhibitor GSK343 (purple), compared with ESCs expressing WT H3.3 (blue). (**D**) Genomic distribution of mapped reads of H3.3 72 h after Dox.

To validate our system, we performed western blots 72 h before and after Dox addition confirming the expression of the exogenous H3.3 without globally altering the levels of endogenous H3 (Supplementary Figure S1C, left). We further validated the expression of H3.3K27M in the mutant cells, and, as expected, observed a global decrease in H3K27me3 and a global increase in H3K27ac in the H3.3K27M-expressing cells (Supplementary Figure S1C, right). We then performed ChIP-Seq 72 h after Dox addition for H3K27me3, H3K27ac and H3, which we used for global normalization (41). We once again observed a global decrease in H3K27me3 and a reciprocal increase in H3K27ac in cells expressing H3.3K27M (Supplementary Figure S1E). To better support our model, we also measured H3K27M and H3K27me3 levels in DIPG/DMG cell culture derived previously from several DIPG/DMG patients (36), as well as in our ESC system. To obtain reliable quantitative data we used Cytometry by Time of Flight (CyTOF) to measure H3.3K27M and H3K27me3 expression levels, as well as the levels of the core histones H3, H4 and H3.3. To overcome technical variability, we normalized H3.3K27M and H3K27me3 expression to the core histone levels in each sample. We profiled the expression levels of three DIPG/DMG patient derived cell lines (DIPG13; DIPG25 and DIPG50), harboring the H3K27M mutation, and mouse ESCs following 4 days of Dox treatment to induce either the WT or the mutant H3.3. Our CyTOF results show comparable levels of H3K27M in the DIPG/DMG lines and the ESC inducible system (Supplementary Figure S1F, G). Moreover, similarly to DIPG/DMG, induction of H3K27M in ESCs leads to a significant reduction in H3K27me3 (Supplementary Figure S1F, blue). These results further support the relevance of our ESC system to study the epigenetic alteration that occur in DIPG. Heatmaps of H3K27ac ChIP-seq around transcription start sites (TSSs) revealed the expected distribution of H3K27ac in both WT and the mutants-expressing lines (Figure 1A). Quantification of H3K27ac levels revealed, as was previously reported (45), that the increase in global H3K27ac in the presence of H3.3K27M results in lower levels of H3K27ac around TSSs and enhancers (Supplementary Figure S1H-I). We also inhibited the PRC2 complex with the small molecule GSK343, which also resulted in the expected increased H3K27ac and decreased H3K27me3 (Supplementary Figure S1J).

We then sought to analyze the genome-wide binding pattern of the induced H3.3 itself and its incorporation. To this end, we once again performed ChIP-seq using HA-specific antibodies 72 h following Dox addition. Heatmaps of H3.3-HA ChIP-seq around TSSs showed the expected binding patterns (Figure 1B), and the metagene profile of H3.3 was as previously observed (15), with a double-peak surrounding the TSS (Figure 1C). The global genomic distribution of the incorporated H3.3 revealed, as expected, enrichment around promoter regions and gene bodies (Figure 1D). While the overall global distribution of H3.3 in the two mutants resembled that of the WT cells, we observed slight but significant increased incorporation around promoters (Figure 1C, D, *P* << 10^−10^, *t*-test, Fisher exact test).

### Enhanced early incorporation of mutated H3.3 in TSS

**Figure 2.**
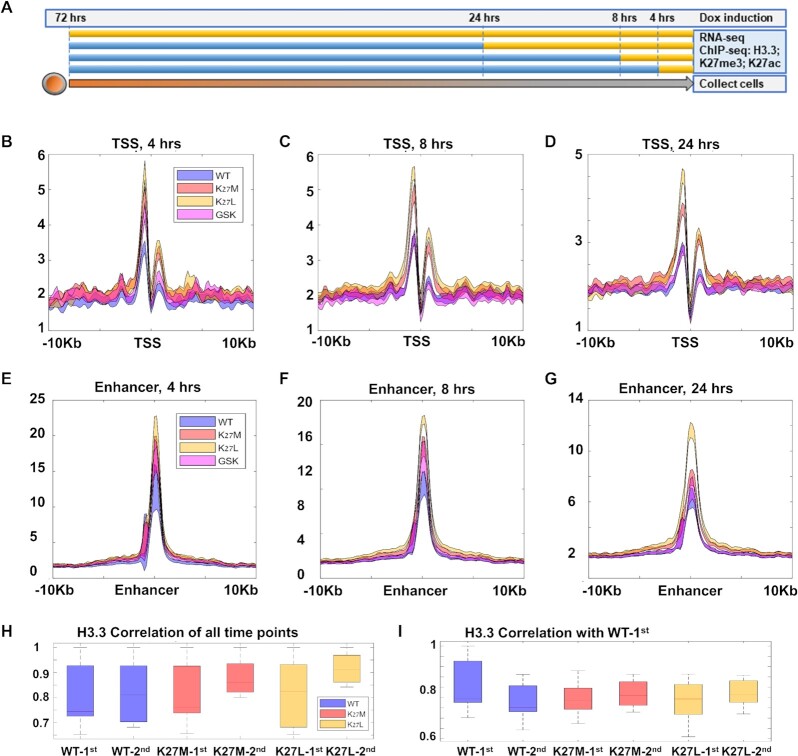
Increased incorporation of both H3.3K27M and H3.3K27L around TSSs and enhancers. (**A**) Experimental layout. Dox was added at the indicated time points. (**B–D**) H3.3 binding around TSSs, shaded within two standard errors at the indicated time points. ESCs expressing H3.3K27M (red) or H3.3K27L (orange) show increased H3.3 binding around TSSs, not observed in ESCs treated with the PRC2 inhibitor GSK343 (purple), compared with ESCs expressing WT H3.3 (blue). (**E–G**) H3.3 binding around enhancers, shaded within two standard errors at the indicated time points. ESCs expressing H3.3K27M (red) or H3.3K27L (orange) show increased H3.3 binding around enhancers, not observed in ESCs treated with the PRC2 inhibitor GSK343 (purple), compared with ESCs expressing WT H3.3 (blue). (**H**) Correlation of H3.3 binding across all time points (4, 8, 24 and 72 h after Dox) in two independent experiments. (**I**) Correlation of H3.3 binding (at 4, 8, 24 and 72 h after dox induction) with WT-1st (ESCs expressing WT-H3.3, first experiment).

We next wished to analyze H3.3 incorporation dynamics. We performed Time-Seq (15) for H3.3 using HA-specific antibodies, collecting samples 4, 8, 24 and 72 h after Dox addition (Figure 2A). Interestingly, H3.3 enrichment around TSSs was significantly increased in both H3.3K27M and H3.3K27L mutant cell lines, beginning at 4 h after Dox induction, and across all later time points (Figure 1C, Figure 2B-D, *P* << 10^−10^, *t*-test). Similar results for both mutant cell lines were observed around enhancers (Figure 2E–G, *P* < 10^−7^, *t*-test; Supplementary Figure S2A). Other regions, e.g. transcription termination sites (TTS), did not show this increase in H3.3 binding (Supplementary Figure S2B), indicating promoter/enhancer specificity.

Since this behavior was true for both mutants and occurs already in early time points after Dox induction, before the global effect on PRC2 is fully achieved (49), it suggests that the increased enrichment of H3.3 around the TSS in the cells expressing the mutant H3.3 variants is caused by the absence of a modifiable lysine in position 27 rather than by the dominant-negative effect of H3.3K27M on the PRC2 complex and a global reduction in H3K27me3. Supporting this idea, repeating the Time-Seq experiments in the presence of the PRC2 inhibitor GSK343 did not affect H3.3 incorporation dynamics in TSS (Figure 2B–D; Figure 1C, compare the WT in blue and the GSK343 in purple graphs), despite the observed global reduction in H3K27me3 and elevated H3K27ac levels (Supplementary Figure S1J), but has a small effect in enhancers (Figure 2E–G). We next tested whether the binding pattern of H3.3 changes over time. To this end, we calculated the correlation between the different time-points and found a very high correlation in two independent experiments which we performed several weeks apart (Figure 2H), suggesting that the TSS targets of the tagged H3.3 are stable over time.

So far, we observed increased enrichment of H3.3 in the two mutant-expressing lines around TSSs. To ask if this increase reflects new promoter regions or increased incorporation in existing promoter regions, we analyzed correlations between the different H3.3 ChIP-seq datasets around TSSs. WT-Mutant correlations were overall similar to WT-WT correlations between the two experiments (Figure 2I). Together, these data suggest that at least up to 72 h after Dox addition, both H3.3K27M and H3.3K27L mutants do not bind new targets, supporting previous observations for H3.3K27M (45), but rather show increased H3.3 binding around existing targets.

### H3.3 mutations strengthen the association between H3.3 binding around active promoters and gene expression

**Figure 3.**
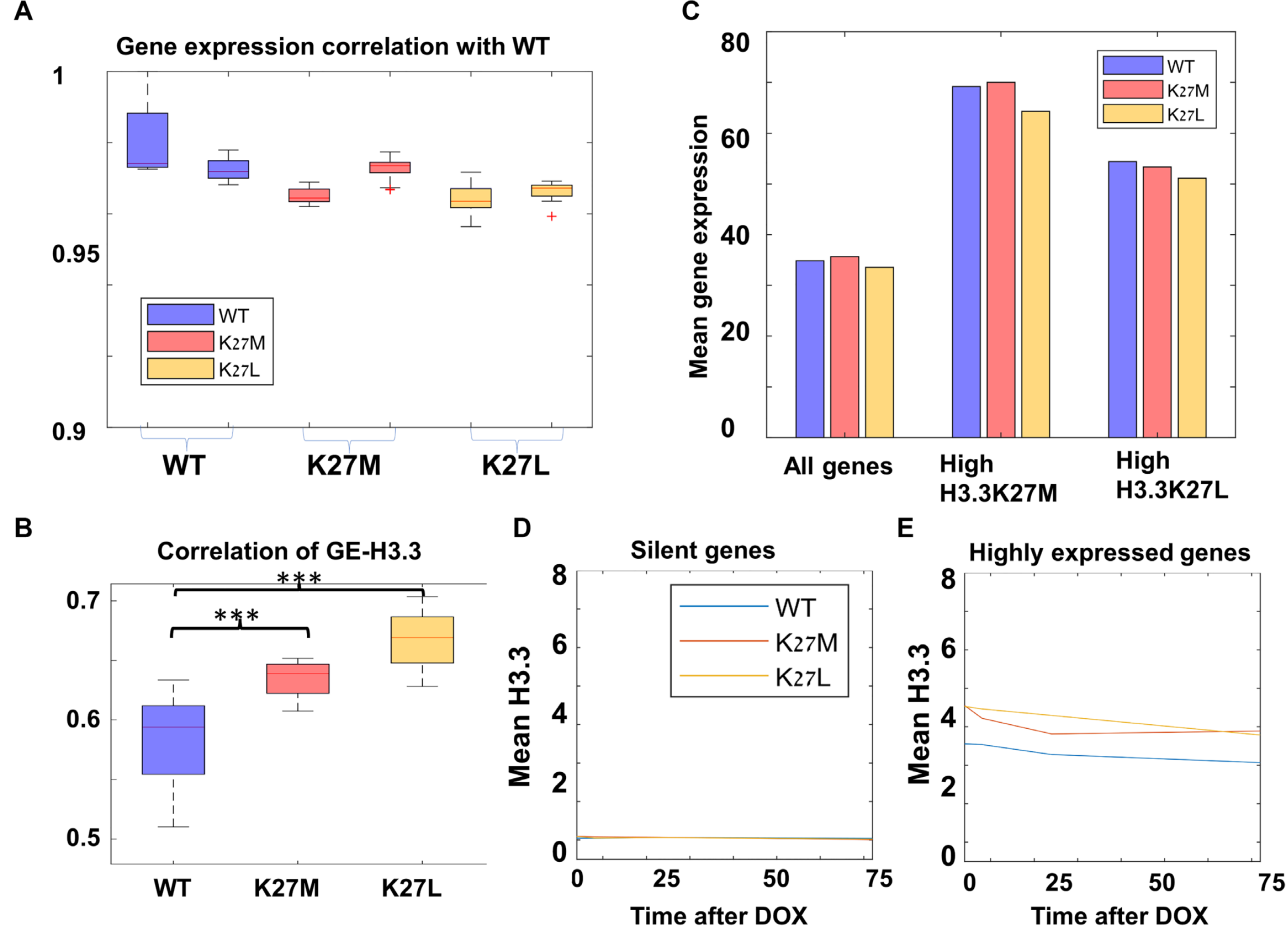
H3.3K27M/L mutations lead to increased correlation between H3.3 binding and gene expression. (**A**) Gene expression correlations between the different lines and WT. (**B**) Correlation between H3.3 incorporation and gene expression. (**C**) Mean gene expression of all genes (left bars), genes enriched with H3.3K27M (middle) or H3.3K27L (right), in ESCs expressing WT-H3.3 (blue), H3.3K27M (red) and H3.3K27L (orange). (**D**, **E**). Mean incorporation of WT-H3.3 (blue), H3.3K27M (red) and H3.3K27L (orange) across the different time-points around silent genes (D) and highly expressed genes (E).

To examine the immediate effects of H3.3K27M and H3.3K27L on gene expression, we harvested the cells at different time points (Figure 2A) following Dox addition (4, 8, 24 and 72 h), and analyzed gene expression by RNA-sequencing (RNA-seq). Overall, we found very little changes in gene expression despite the global reduction in H3K27me3 after 72 h in the H3.3K27M expressing cells. Gene expression patterns were highly correlated (>0.9, Spearman Correlation) between the WT cells and the cells expressing the mutated H3.3 variants across all different time points following Dox addition (Figure 3A), and gene expression data of WT and H3.3K27M did not cluster separately (tSNE, Supplementary Figure S2C). Overall, these results suggest that gene expression patterns are mostly conserved between WT and mutated H3.3 lines, at least during the first days following the expression of the mutation.

Despite these global similarities, several specific genes showed reproducible upregulation after 24 and 72 h of H3.3K27M expression. These include *Vegfa*, which encodes a growth factor responsible for proliferation and migration of vascular endothelial cells (51); *Ddit4*, which encodes a protein regulating cell proliferation and survival, inhibition of neuronal differentiation and neurite outgrowth, and might generate resistance to cancer therapy (52,53); *Grin1*, which encodes a critical subunit (zeta) of the NMDA receptor, and *Bhlhe40*, which encodes a transcriptional repressor regulating the activity of clock related genes that might play a role in cancer (54) (Supplementary Figure S3A). Comparing our results with available RNA-seq datasets from human NPCs derived from brainstem of 19-weeks-old embryos expressing exogenous v5-H3.3K27M (45), we found elevation of some of those genes (*A2m*, *Bhlhe40* and *Ier3*) in the H3.3K27M-expressing lines (Supplementary Figure S3B).

As expected, since H3.3 is preferentially enriched around active genes, H3.3 binding correlated with expression. Interestingly, ESCs expressing H3.3K27M or H3.3K27L showed a significantly higher correlation of H3.3 incorporation with gene expression than cells expressing WT H3.3 (Figure 3B, *P* < 0.00005, *t*-test). In addition, both mutants displayed stronger correlations (negative or positive, respectively) between gene expression and H3K27me3 or H3K27ac (Supplementary Figure S3C-D, *P* < 0.02, *t*-test). Since this was true for both mutants, it cannot be explained by the dominant-negative effects on PRC2.

Next, we closely inspected the association between gene expression and H3.3 incorporation around gene promoters. We found that genes with higher levels of mutated H3.3 are highly expressed in all lines (Figure 3C), suggesting again that mutated H3.3 does not bind new targets, but instead binds active genes more efficiently, or is evicted more slowly. We then analyzed separately H3.3 binding around genes according to expression level. As expected, H3.3 binding was low in the lowly-expressed genes and high in the highly-expressed genes across all cell lines. However, in the highly-expressed genes, H3.3 binding was higher in cells expressing the mutant H3.3 variants (Figure 3D, E). This suggests that increased incorporation of mutant H3.3 occurs preferentially in highly-expressed genes, as can also be observed in differentially expressed genes (Supplementary Figure S3E–H).

### H3.3K27 mutations disrupt gene expression patterns during differentiation

**Figure 4.**
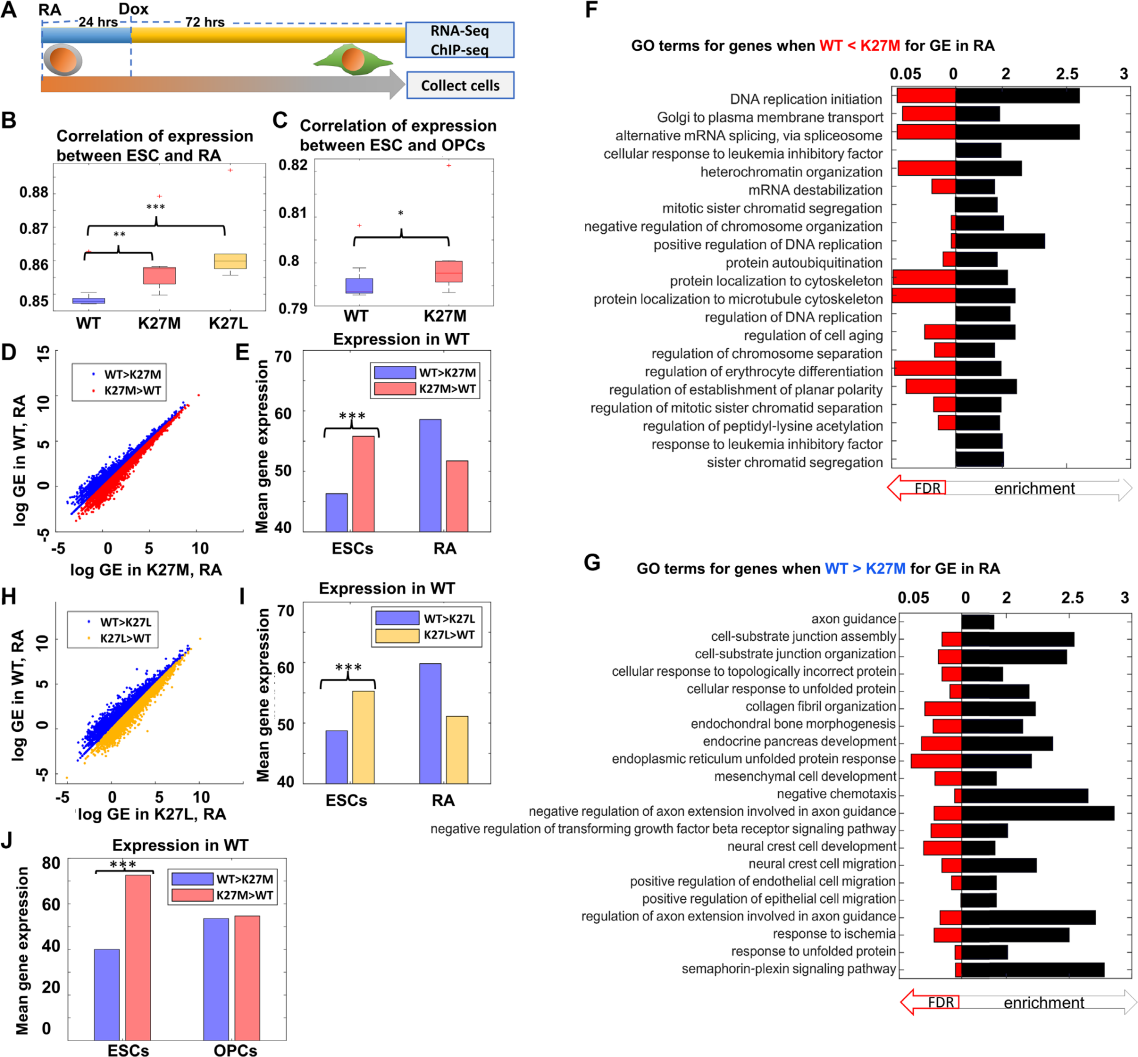
Expression of H3.3K27/L in differentiated ESCs support the retention of ESC-expressed genes. (**A**) Experimental layout. (**B**) Correlation of gene expression between ESCs and RA treated cells. ESCs expressing both H3.3K27M (red) and H3.3K27L (orange) mutants exhibit higher correlation with the undifferentiated state compared with ESCs expressing WT-H3.3 (blue) (**P < 10^−3^; ***P < 10^−4^). (**C**) Same as B in ESC-derived OPCs (*P < 0.05). (**D**) Log gene expression values in RA-induced ESCs, comparing ESCs expressing WT-H3.3 (Y-axis) with ESCs-expressing H3.3K27M (X-axis). Blue and red denote genes higher in RA treated ESCs expressing H3.3-WT and RA treated ESCs expressing H3.3K27M, respectively. (**E**) Quantification of gene expression differences in ESCs in 2i condition, of gene groups defined by activity in RA (D) (***P < 10^−50^). (**F**) Most enriched GO terms for genes higher in ESCs expressing H3.3K27M versus ESCs expressing WT-H3.3, in RA-induced ESCs. (**G**) Same as (F) for genes higher in ESCs expressing WT-H3.3 versus ESCs expressing H3.3K27M, in RA-induced ESCs. (**H, I**) Same as (D, E) for ESCs expressing H3.3K27L (I: ***P < 10^−50^). (**J**) Same as (E) in ESCs and OPCs (***P < 10^−5^).

We next asked if the induction of H3.3K27M/L may affect gene expression during differentiation. To this end, we differentiated our ESCs using retinoic acid (RA, 1 μM, 4 days). We added Dox 24 h after the onset of differentiation, and after 72 h (completing 96 h of differentiation) performed RNA-seq (Figure 4A). Within the differentiated populations, we saw no major global differences in gene expression between the WT and the mutant cells (Spearman's correlation ∼0.96). In contrast, the correlation between undifferentiated and RA-induced cells was significantly higher in both the H3.3K27 mutants compared with WT cells (Figure 4B, *P* < 0.001, *t*-test). This may suggest that both H3.3K27 mutant lines remain closer to the undifferentiated state and fail to fully repress previously active genes and/or induce the necessary gene expression programs required for early RA-induced differentiation. To further test the validity and relevance of these findings to DMG, we differentiated the WT and the H3.3K27M ESC clones into oligodendrocyte progenitor cells (OPCs), which are considered to be the cell of origin of DMG (55,56). Again, we found that in OPCs, the cells expressing H3.3K27M showed a higher gene expression correlation with undifferentiated ESCs than the WT OPCs (Figure 4C, *P* < 0.05, *t*-test).

To test this more directly and to examine the gene expression signature associated with the increased correlation observed in the mutant cells, we analyzed genes in the differentiated cells, that were highly expressed in the H3.3K27M mutants compared to WT (Figure 4D) and vice versa. We found that genes which had higher expression in the mutant differentiated cells are, as a group, highly expressed in ESCs even in WT cells (Figure 4E, *P* << 10^−10^, *U*-test), as well as, as expected, in the mutants (Supplementary Figure S4A). Gene Ontology (GO) revealed that the group of genes, which are higher in the H3.3K27M expressing cells, are related to DNA replication and repair, and other mitotic related processes (Figure 4F), while genes which were higher in the WT cells are related to neural differentiation (Figure 4G). This effect was observed in both H3.3K27M and H3.3K27L mutants (Figure 4H-I, *P* << 10^−10^; Supplementary Figure S4B-D), suggesting that it is a result of loss-of-function (the absence of lysine) rather than a gain-of-function (inhibition of PRC2) effect. Many of the genes which were expressed at higher levels in the H3.3K27M-expressing differentiated cells compared with WT were also higher in the H3.3K27L-expressing cells, and vice versa (>50% of genes), suggesting a shared, PRC2-independent, mechanism between the two mutations. These genes included many of the *bona fide* pluripotency-related genes including *Sox2*, which was observed in cancer cells derived from patients (36,49) and *Nanog* (Supplementary Figure S4E-J).

Similar analysis in OPCs further confirmed these findings. We found that genes which were more active in the H3.3K27M OPCs than WT cells, were already expressed at higher levels in undifferentiated ESCs expressing the mutant H3.3 (Figure 4J, *P* < 10^−5^, *U*-test). Here again we find that those genes which were more active in H3.3K27M OPCs compared to WT OPCs are related to DNA replication (Supplementary Figure S4K), strengthening the idea that incorporation of mutant H3.3 favors a stem cell-like state also in OPCs. Repeating the same analysis with a cutoff of 1.5 yielded similar results (Supplementary Figure S5A–C), with a stronger GO enrichment of DNA replication (Supplementary Figure S5D).

### H3.3 incorporation affects gene expression

**Figure 5.**
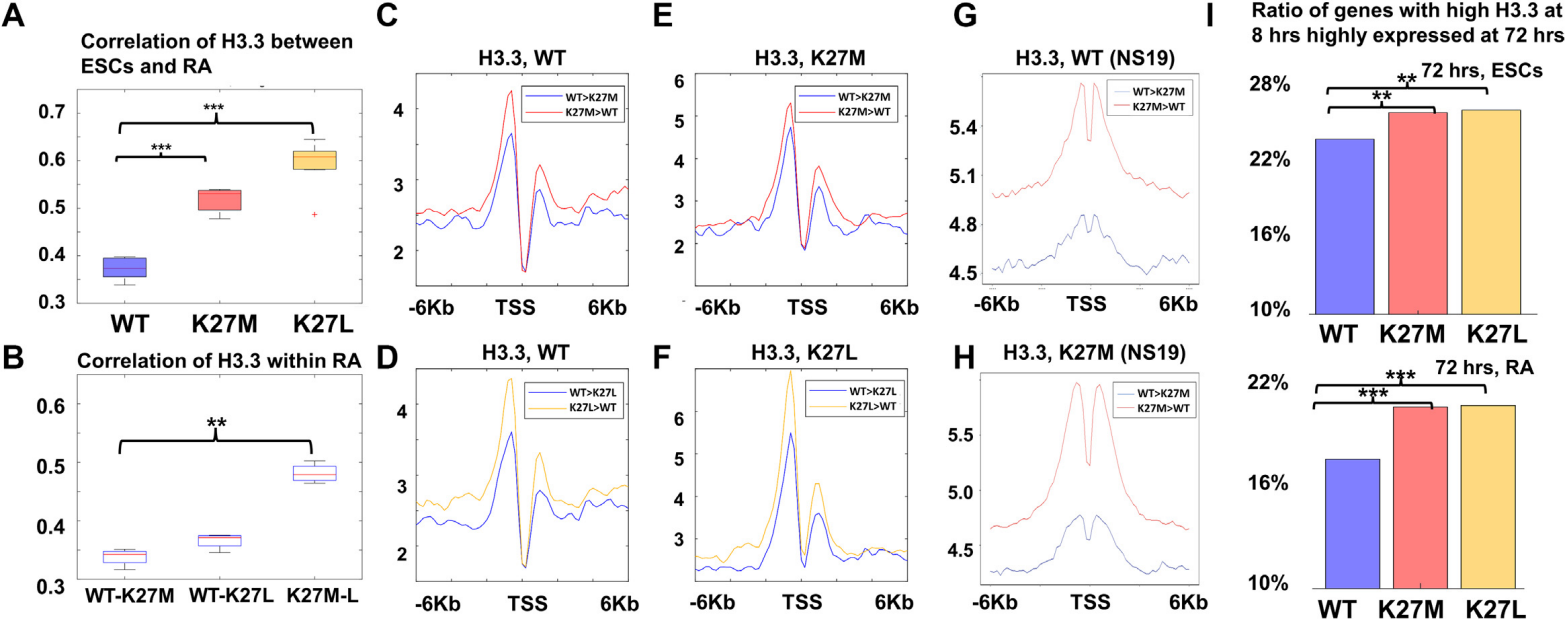
Mutant cells differentiated to neural progenitors retain the expression of genes which are highly expressed in the pluripotent state. (**A**) Correlation of H3.3 incorporation around TSSs between ESCs and RA-induced cells in ESCs expressing WT-H3.3 (blue), ESCs expressing H3.3K27M (red) and ESCs expressing H3.3K27L (orange) (***P < 10^−5^). (**B**) Correlation of H3.3 incorporation around TSSs between ESCs expressing WT-H3.3 and ESCs expressing H3.3K27M (left); ESCs expressing WT-H3.3 and ESCs expressing H3.3K27L (middle), and ESCs expressing H3.3K27M and ESCs expressing H3.3K27L (right) (**P < 10^−3^), in RA-induced ESCs. (**C**) H3.3 meta-gene enrichment plots in WT ESCs around TSSs of genes expressed higher in the RA-treated H3.3K27M cells compared to WT-H3.3 cells (red) and vice versa (blue). (**D**) H3.3 meta-gene enrichment plots in WT ESCs around TSSs of genes expressed higher in the RA-treated H3.3K27L cells compared to WT-H3.3 cells (yellow) and vice versa (blue). (**E**) H3.3 meta-gene enrichment plots in H3.3K27M-expressing ESCs around TSSs of genes expressed higher in the RA-treated H3.3K27M cells compared to WT-H3.3 cells (red) and vice versa (blue). (**F**) H3.3 meta-gene enrichment plots in H3.3K27L-expressing ESCs around TSSs of genes expressed higher in the RA-treated H3.3K27L cells compared to WT-H3.3 cells (yellow) and vice versa (blue). (**G**) H3.3 meta-gene enrichment plots in human embryo-derived cells (NS19) expressing WT-H3.3 from Brien et al (45) around TSSs of genes higher in human embryo-derived cells expressing WT-H3.3 compared with human embryo-derived cells expressing H3.3K27M (blue) and vice versa (red). (**H**) Same as (G) in human embryo-derived cells expressing H3.3K27M. (**I**) Ratio of High-H3.3-at-8-h-genes expressed at 72 h in ESCs expressing WT-H3.3 (blue), ESCs expressing H3.3K27M (red) and ESCs expressing H3.3K27L (orange). Top: in undifferentiated ESCs (**P < 0.005); Bottom: in RA-induced ESCs (***P < 10^−5^).

To ask whether the gene expression signature of delayed differentiation is related to H3.3 incorporation itself, we next compared the H3.3 binding maps between ESCs and RA-induced cells in our different cell lines (WT, H3.3K27M, H3.3K27L). In both lines expressing mutant H3.3, the RA-treated cells showed a higher correlation (Spearman) with the ESCs compared with cells expressing WT H3.3 (Figure 5A, *P* < 10^−5^, *t*-test). Also, H3.3 binding in the cells expressing H3.3K27M was more similar to the cells expressing H3.3K27L than to cells expressing WT H3.3, suggesting, once again, a shared mechanism, independent of PRC2 (Figure 5B, *P* < 10^−3^, *t*-test). Similar increase, though to overall lower correlation between RA-treated cells and ESCs, was observed in enhancers (Supplementary Figure S6A, B, *P* < 0.05).

We then analyzed H3.3 incorporation around the genes whose expression was elevated in either the mutant cells or the WT cells following RA induction. Genes which were elevated in the mutant cells had higher level of H3.3 incorporation than genes which were elevated in the WT cells. This was true for all cells: WT (Figure 5C, D), H3.3K27M (Figure 5E) and H3.3K27L (Figure 5F), suggesting again that the genes which their expression is altered after differentiation are those genes which are active in stem cells, and therefore have higher incorporation of H3.3. These results link H3.3 incorporation in ESCs with higher gene expression in cells expressing mutant H3.3 after differentiation, suggesting impaired regulation of gene expression during differentiation of genes bound by mutated H3.3.

We also analyzed data from human embryo-derived cell lines, from Brien *et al.* (45) and calculated the H3.3 signature around genes, which were up- or down-regulated in the H3.3K27M cells, and, once again, found that the elevated genes in the H3.3K27M cells had higher levels of H3.3 incorporation around their TSSs (Figure 5G, H, Supplementary Figure S6C, D).

Finally, we tested the influence of H3.3 binding on later expression. We asked if increased binding of H3.3 around a TSS in an earlier time point is an indication for higher expression of the bound gene in a later time point. Comparing WT and mutant-expressing cells at the 8 h and the 72 h time points, we found that in the mutant cells, a significantly higher (*P* < 0.003, Fisher's exact test) proportion of genes with increased H3.3K27M/L binding at 8 h, were highly expressed at the 72 h time point, compared with WT-H3.3-bound genes (Figure 5I, top, *P* < 0.005). This effect was stronger in the early differentiating, RA-treated cells (Figure 5I, bottom, *P* < 10^−5^). These results suggest that H3.3 incorporation around gene promoters influences the expression level of the incorporated genes.

## DISCUSSION

DIPG/DMG is one of the deadliest tumors with a life expectancy of less than a year. In the majority of the DMG cases it is caused, at least in part, by H3.3K27M. The K-to-M substitution, apart from disabling lysine methylation was also shown to inhibit the PRC2 complex, thus acting in a dominant-negative fashion. Here we aimed to explore the immediate consequences of ectopic expression of H3.3K27M, and to tease apart effects caused by the loss of lysine methylation versus PRC2 inhibition. Using Dox-inducible expression of either WT, K27M or K27L versions of H3.3 in ESCs and during ESC differentiation, we show that expression of mutant H3.3 in differentiating ESCs leads to an immature gene expression signature, which corresponds to the binding signature of the mutated histone. Interestingly, we observed a similar immature expression and epigenetic signature in cells expressing H3.3K27L, suggesting that these immediate consequences of the mutated histone are PRC2-independent.

Using Time-Seq experiments (15), we also analyzed H3.3 incorporation across the genome and show that H3.3K27M binds TSSs more strongly than WT. Once again, we observed the same phenomenon for H3.3K27L, suggesting that K27 modifications, and not PRC2 activity, are required for H3.3 turnover around gene promoters. Interestingly, recent proteomic studies comparing proteins specifically associated with either the WT or the H3.3K27M mutant, identified several histone chaperones, including ASF1B and FACT complexes (39), which were found to associate with H3.3K27M but not with WT-H3.3, providing a potential mechanism explaining our observations of differential incorporation dynamics.

As expected, chromatin incorporation of H3.3 correlates with gene expression, as previously shown (14,15,57). Interestingly, we find that during differentiation, H3.3 incorporation at early time-points predicts expression at later time-points. While this was true for both WT and mutant forms of H3.3, both H3.3K27M and H3.3K27L mutants displayed a stronger predictive power than WT H3.3, suggesting that increased presence of H3.3 in the mutants, might be directly acting to promote gene expression.

It was previously shown that pervasive H3K27 acetylation in DMG-derived tumor cell lines leads to aberrant expression of endogenous retroviruses (ERVs) across the genome (58), operating as an anti-tumor immune response defense mechanism. Treatment with histone deacetylase inhibitors (HDACi) further exacerbated this response, resulting in increased vulnerability of the tumor cells and their accelerated elimination. Our work further suggests that K27 mutations promote a stem-like state. Since HDACi generally accelerate stem cell differentiation (59–61), the treatment of cells with HDACi could potentially confer vulnerability to DMG cells by multiple pathways.

Combined, our findings suggest that impaired dynamics of mutant H3.3 keeps active genes active for a longer time (Supplementary Figure S7). This, in turn, slows differentiation, potentially explaining the immediate consequences of the H3.3K27M mutation, independent of processes related to PRC2 inhibition. These findings are in line with previous reports which highlight the necessity of residual PRC2 activity for proliferation (26) as well as more recent data which suggest that existing PRC2 domains are not the direct targets of H3.3K27M (56,62,63). Our findings provide important insights regarding the mechanisms by which H3.3K27M might be causing aggressive tumors in children and raises caution when considering drugs which are designed solely to reactivate PRC2 to restore normal cellular activity, as potential therapy. Our data suggest that the absence of a modifiable H3.3 leads to a gene expression signature reminiscent of stem-like cells, which might contribute to uncontrolled proliferation of the mutation-bearing cells, and that targeting the PRC2-dependent effect alone may not suffice to treat this devastating disease.

## DATA AVAILABILITY

The sequencing data from the study have been submitted to the NCBI Gene Expression Omnibus (GEO), accession number GSE195789.

## Supplementary Material

gkac800_Supplemental_File
