# Correction to ‘PRC2-independent actions of H3.3K27M in embryonic stem cell differentiation’

**DOI:** 10.1093/nar/gkad018

**Published:** 2023-01-10

*Nucleic Acids Research*, 2022; gkac800, https://doi.org/10.1093/nar/gkac800

In the originally published version of this manuscript, there was a typographical error in the title, whereby ‘PRC2-independent’ was incorrectly given as ‘PRC2-indepdendent’.

This error has been corrected.

