# Supplementary material for: PRC2-independent actions of H3.3K27M in embryonic stem cell differentiation
Source: Nucleic Acids Res. 2022 Sep 26;51(4):1662–73. doi: 10.1093/nar/gkac800 (PMC9976889; doi:10.1093/nar/gkac800)
Supplement: gkac800_Supplemental_File [file gkac800_supplemental_file.pdf]

Supplementary Figures and Figure Legends

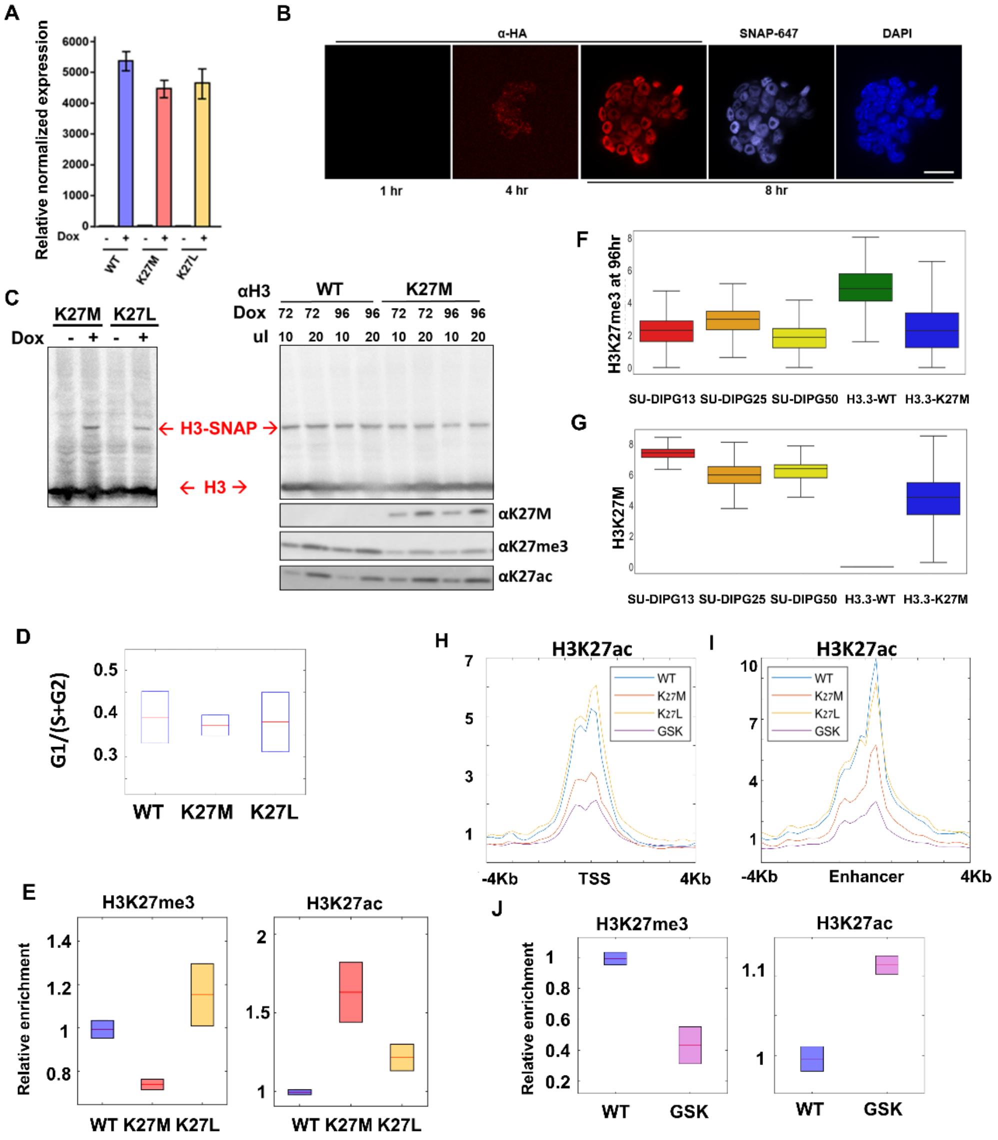

Supplementary Figure 1. Experimental system validation.

(**A**) RT-qPCR before (-) and after (+) Dox addition in ESCs expressing WT-H3.3 (gray), H3.3K27M (red) or H3.3K27L (orange). (**B**) Immunofluorescence (IF) images of ESCs expressing H3.3K27M using α-HA antibodies (red) at the indicated time-points after Dox. H3.3 is visible at the 4hr time-points and is expressed at high levels in all cells at the 8 hr time-point. Purple: SNAP-647 ligand; Blue: DAPI. Scale bar = 50 µm. (**C**) Left: Western blots for histone H3 before (-) and after (+) Dox addition in ESCs expressing either H3.3K27M (left) or H3.3K27L (right). Right: Western blots for histone H3, H3K27M, H3K27me3 and H3K27ac, 72 or 96 hrs after Dox (10 or 20 µl indicate the amount of sample). (**D**) Cell cycle analysis (average of two independent experiments) in ESCs expressing WT-H3.3 (left), H3.3K27M (middle) and H3.3K27L (right) for 24 hours. Y-axis denotes the G1/S ratio. Changes are non-significant. (**E**) Global levels of H3K27me3 (left) and H3K27ac (right) in ESCs expressing WT-H3.3, H3.3K27M (red) and H3.3K27L (orange). Score was calculated as total read count normalized to H3, then re-renormalized to the 4 and 8 hr time-point. (**F**) H3K27me3 levels, as measured by Cytometry by Time of Flight (CyTOF), of three DIPG cell lines (SU-DIPG13, SU-DIPG25 and SU-DIPG50 labeled red, orange and yellow, respectively) carrying the H3-K27M mutation, as well as our mouse ESCs following 4 days of induction of WT (green) or mutant (blue) H3.3 expression. Presented are expression levels following transformation, scaling and normalization. (**G**) same as (F), for H3.3K27M. (**H**) Average plot of H3K27ac levels around transcription start sites (TSS) of H3-WT (blue), H3-K27M (red), H3-K27L (yellow) and upon GSK treatment (purple) at 72 hours after Dox. (**I**) Average plot of H3K27ac levels around enhancers of H3-WT (blue), H3-K27M (red), H3-K27L (yellow) and upon GSK treatment (purple) at 72 hours after Dox. (**J**) Same as (**D**) comparing untreated WT ESCs to WT ESCs treated with the PRC2 inhibitor GSK343.

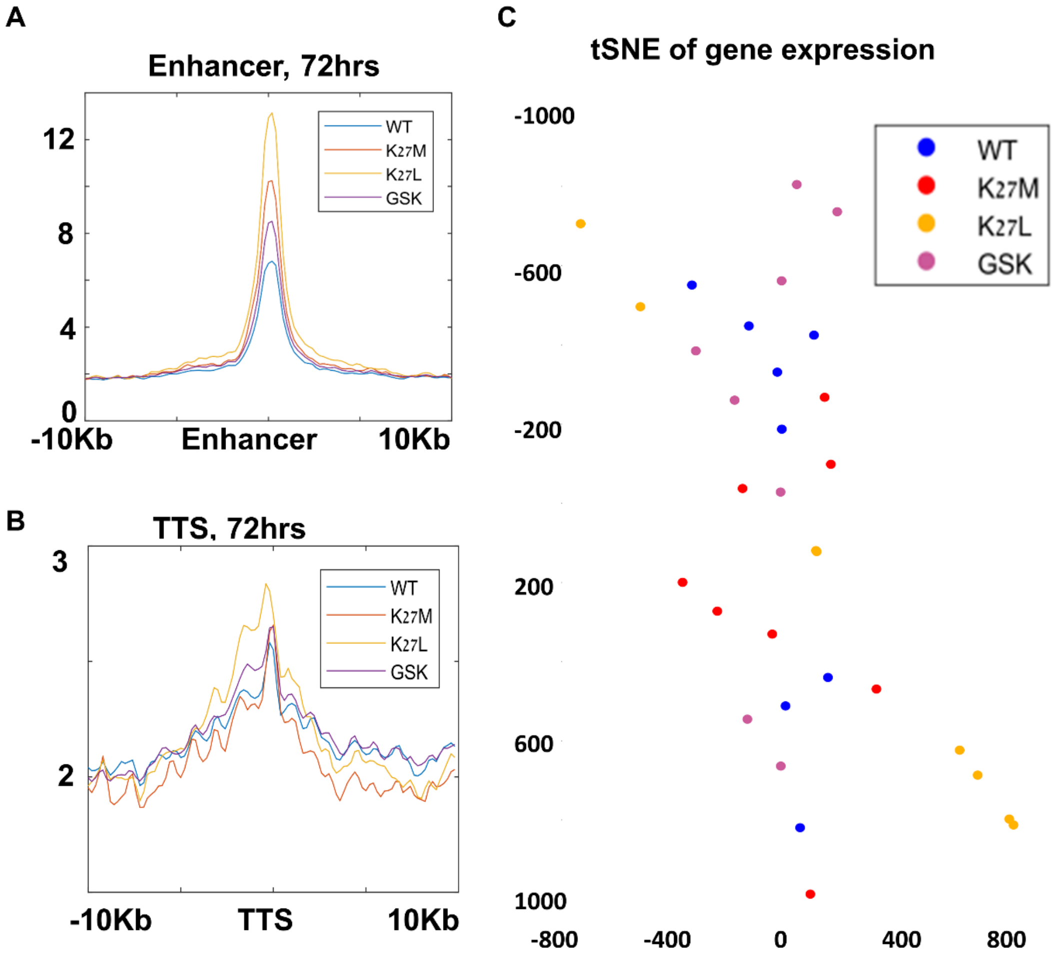

Supplementary Figure 2. H3.3 incorporation and global gene expression in ESCs expressing WT or mutant H3.

(**A**) H3.3 binding around enhancers at 72 hours after Dox In ESCs expressing WT-H3.3 (blue), H3.3K27M (red), H3.3K27L (orange), and in ESCs treated with the PRC2 inhibitor GSK343 (purple). (**B**) Same as (A) around transcription termination sites (TTS). (**C**) tSNE of gene expression data of ESCs expressing WT-H3.3 (blue), H3.3K27M (red), H3.3K27L (orange) and ESCs treated with GSK343 (purple).

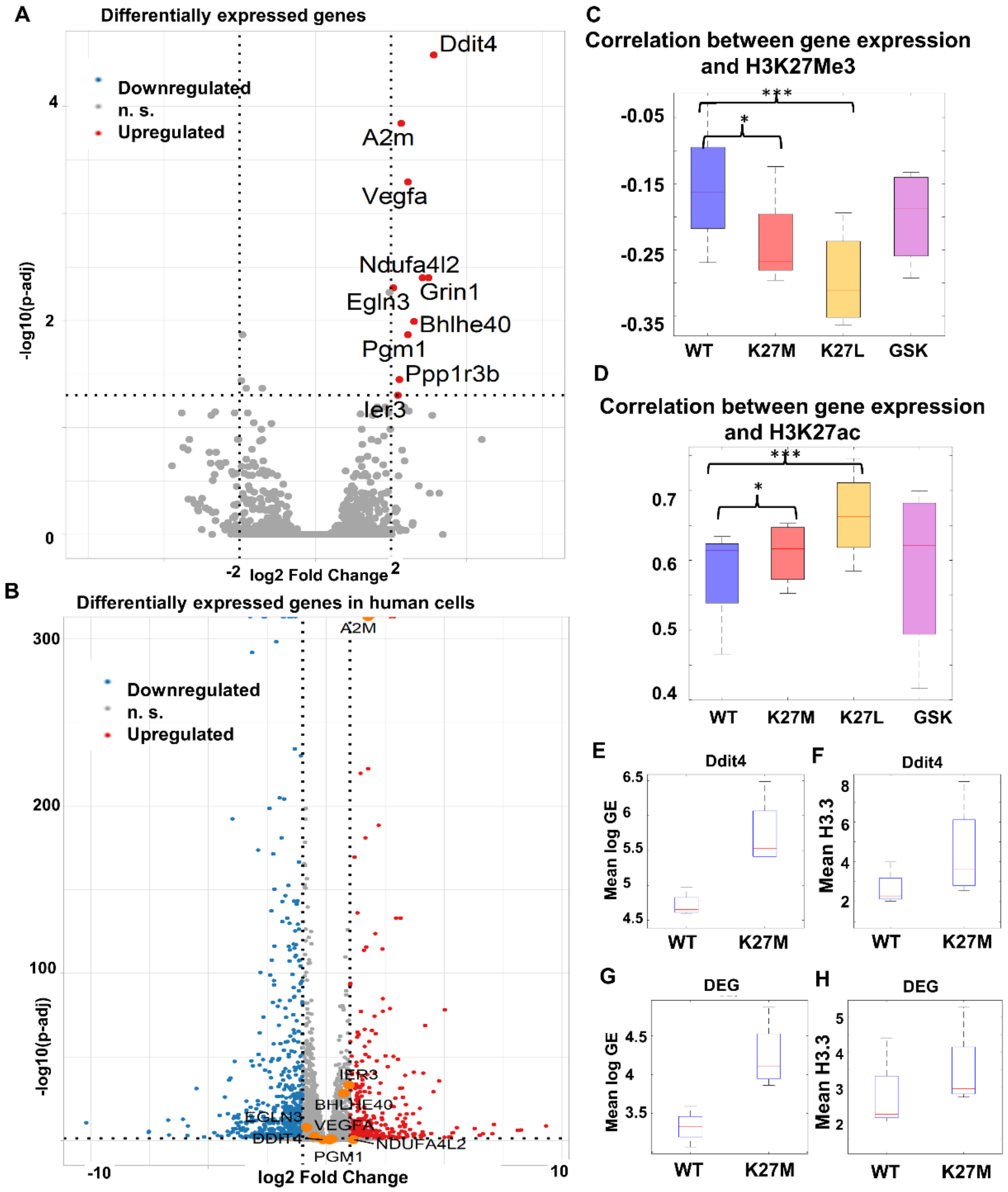

Supplementary Figure 3. Gene expression correlation between the different line.
(**A**) Volcano plot of differentially expressed genes between ESCs expressing WT-H3.3 and ESCs expressing H3.3K27M, 72 hrs after Dox. Significantly upregulated genes are marked in red. **(B)** Volcano plot of differentially expressed genes between human fetal neurons expressing WT-H3.3 or H3.3K27M from Brien et al (37). Significantly up- and down-regulated genes are marked in red and blue, respectively. Genes upregulated in mESCs are marked in orange. (**C**) Correlation between gene expression and H3K27me3 In ESCs expressing WT-H3.3 (blue), H3.3K27M (red), H3.3K27L (orange) and ESCs treated with GSK343 (purple). (Asterisks: *, $P < 0.02$; *** $P < 10^{-5}$). (**D**) Same as (D) for H3K27ac. (Asterisks: *, $P < 0.02$; *** $P < 10^{-6}$). **(E)** Mean log expression level of Ddit4 in ESCs expressing WT-H3.3 and H3.3K27M .**(F)** Mean H3.3 enrichment around Ddit4 promoter in ESCs expressing WT-H3.3 and H3.3K27M .**(G)** Mean log expression level of all Differentially Expressed Genes (DEG) in ESCs expressing WT-H3.3 and H3.3K27M. **(H)** Mean H3.3 enrichment around DEG promoters in ESCs expressing WT-H3.3 and H3.3K27M.

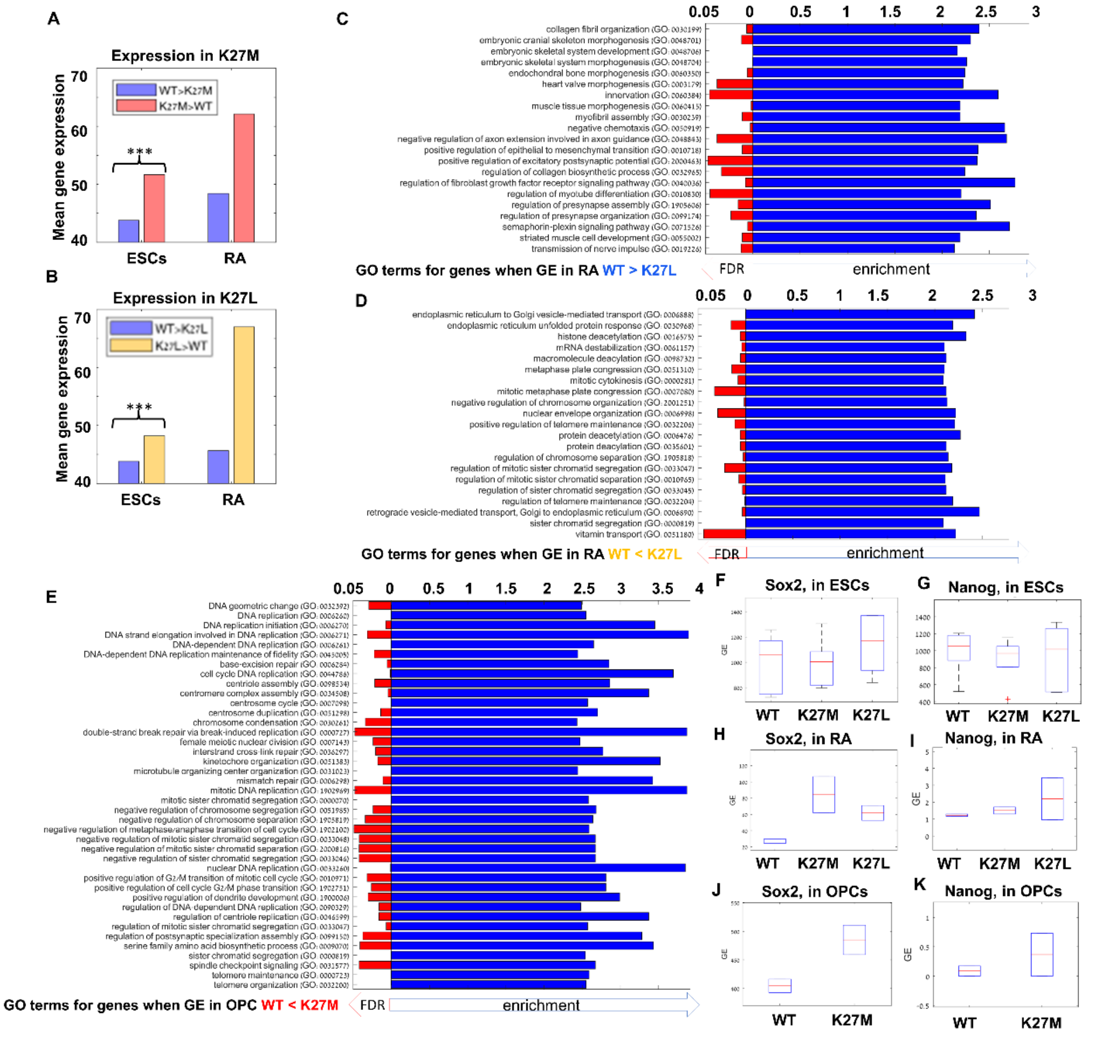

Supplementary Figure 4. Differentiated ESCs expressing H3.3K27M or H3.3K27L retain expression of ESC-expressed genes.

(**A**). Quantification of gene expression differences between gene groups defined by activity in RA-treated ESCs (defined in A) in undifferentiated ESCs expressing K27M-H3.3. (**B**) Quantification of gene expression differences between gene groups defined by activity in RA-treated ESCs (defined in A) in undifferentiated ESCs expressing K27L-H3.3. (**C**) Most enriched GO terms for genes higher in ESCs expressing WT-H3.3 versus ESCs expressing H3.3K27L, in RA-induced ESCs. (**D**) Most enriched GO terms for genes higher in ESCs expressing H3.3K27L versus ESCs expressing WT-H3.3, in RA-induced ESCs. (**E**) Most enriched GO terms for genes higher in ESCs expressing H3.3K27M versus ESCs expressing WT-H3.3, in ESC-derived OPCs. (**F-K**) Expression level of Sox2 (F, H, J) and Nanog (G, I, K) in ESCs (F-G), RA-treated ESCs (H-I) and OPCs (J-K) in WT-H3.3, H3.3K27M and H3.3K27L.

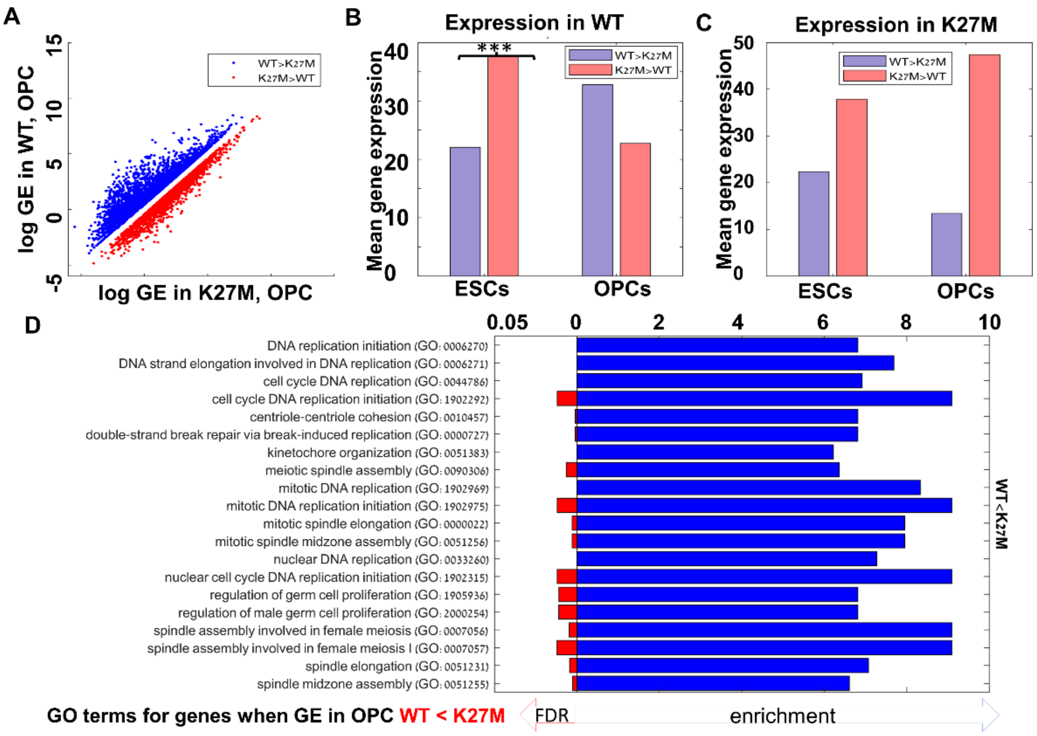

Supplementary Figure 5. Mutant OPCs retain the expression of genes which are highly expressed in the pluripotent state.

(**A**) Log gene expression values of genes >1.5-fold change, comparing OPCs expressing WT-H3.3 (Y-axis) with OPCs expressing H3.3K27M (X-axis). Blue and red denote genes higher in OPCs expressing H3.3-WT and OPCs expressing H3.3K27M, respectively. (**B**) Quantification of gene expression differences between gene groups by activity in OPCs (defined in A) in undifferentiated ESCs expressing WT-H3.3. (**C**) Quantification of gene expression differences between gene groups (defined in A) in undifferentiated ESCs expressing K27M-H3.3. (**D**) Most enriched GO terms for genes higher in OPCs expressing H3.3K27M versus OPCs expressing WT-H3.3.

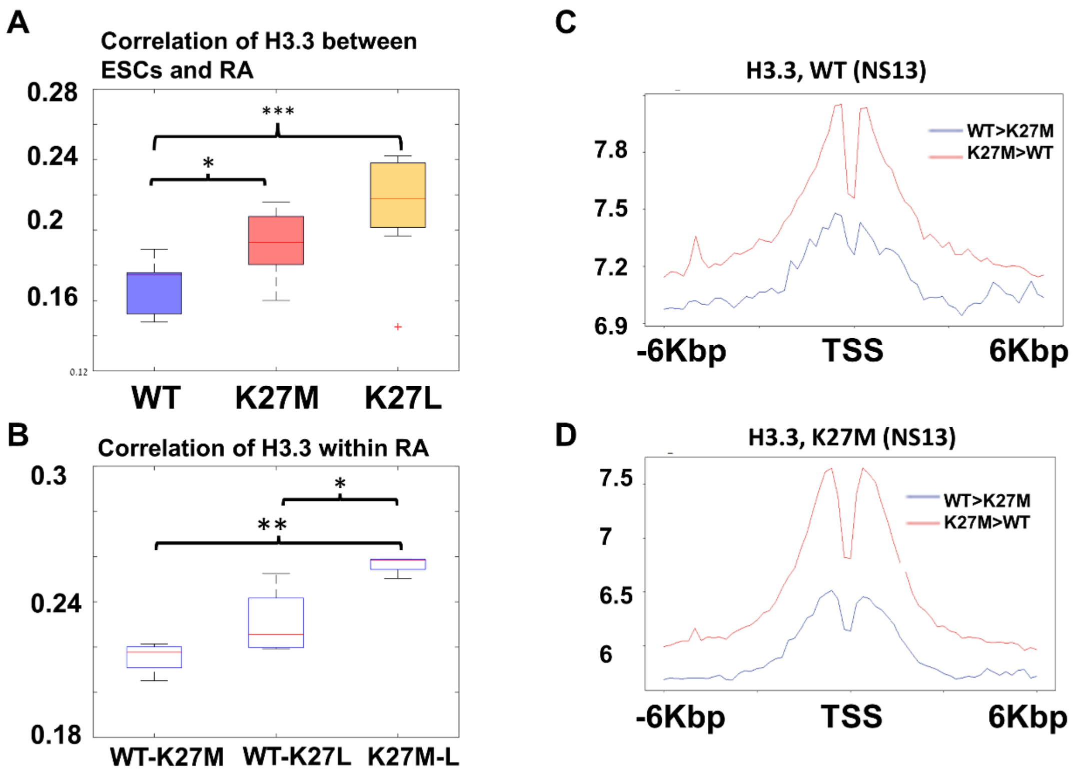

Supplementary Figure 6. H3.3 incorporation predicts gene expression, especially in ESCs expressing mutant H3.3.
(**A**) Correlation of H3.3 incorporation around enhancers between ESCs and RA-induced cells in ESCs expressing WT-H3.3 (blue), ESCs expressing H3.3K27M (red) and ESCs expressing H3.3K27L (orange). (Asterisks: ***, $P < 0.01$; *, $P < 0.05$). (**B**) Correlation of H3.3 incorporation around enhancers between ESCs expressing WT-H3.3 and ESCs expressing H3.3K27M (left); ESCs expressing WT-H3.3 and ESCs expressing H3.3K27L (middle), and ESCs expressing H3.3K27M and ESCs expressing H3.3K27L (right). (Asterisks: **, $P < 0.0001$; *, $P < 0.05$). (**C**) H3.3 meta-gene enrichment plots in human embryo-derived cells (NS13) expressing WT-H3.3 from Brien et al (37) around TSSs of genes higher in human embryo-derived cells expressing WT-H3.3 compared with human embryo-derived cells expressing H3.3K27M (blue) and vice versa (red). (**D**) Same as (C) in human embryo-derived cells expressing H3.3K27M.

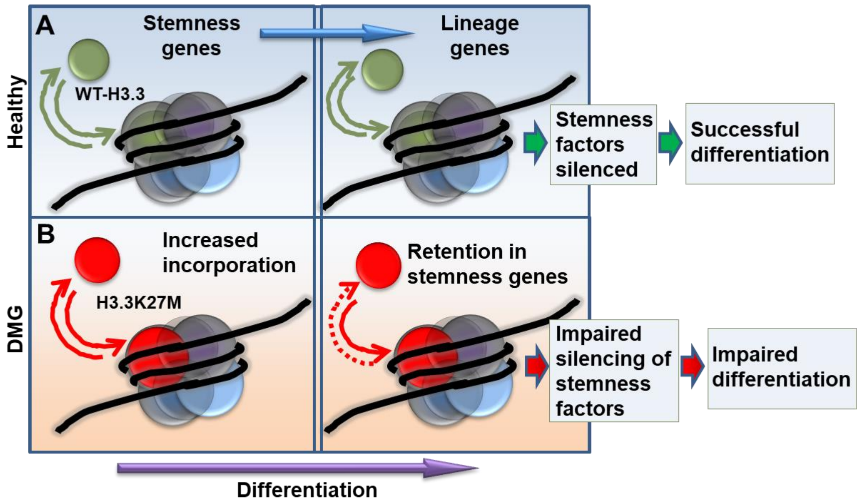

Supplementary Figure 7. A proposed model for non-PRC2 related action of H3.3K27M mutations in DIPG: Loss of K27 leads to impaired H3.3 regulation and incorporation
(A) Turnover of H3.3 is essential for proper differentiation. (B) lack of modifiable K27 interferes with H3.3 turnover, leading to impaired silencing of previously active stemness genes in differentiated cells expressing H3.3K27M.
